# Correlation between circulating lipoprotein(a) levels and cardiovascular events risk in patients with type 2 diabetes

**DOI:** 10.1016/j.heliyon.2024.e37415

**Published:** 2024-09-04

**Authors:** Jun-Xu Gu, Juan Huang, Kun Wang, Yue Yin, Jun-Ling Fang, Ai-Min Zhang, Shan-Shan Li, Xiao-Qin Yao, Ming Yang, Na Zhang, Mei Jia, Ming Su

**Affiliations:** a Department of Clinical Laboratory, Peking University People's Hospital, Beijing, PR China; b Department of Traditional Chinese Medicine, Peking University International Hospital, Beijing, PR China; c Department of Clinical Laboratory, Xiangya Hospital, Central South University, Changsha, PR China; d Department of Clinical Laboratory, Shunyi District Shunan Hospital, Beijing, PR China; e Department of Clinical Laboratory, Peking University International Hospital, Beijing, PR China

**Keywords:** Type 2 diabetes mellitus, lipoprotein(a), Cardiovascular events, Risk indicator, Coronary heart disease

## Abstract

**Background:**

High circulatory lipoprotein(a) [Lp(a)] concentration promotes atherosclerosis; however, its efficacy in predicting the extent of atherosclerotic coronary heart disease (CHD) with coronary artery obstruction and major adverse cardiovascular events (MACEs) in diabetic patients remains questionable. This study aimed to examine whether elevated circulating Lp(a) levels exacerbate CHD and to assess their utility in predicting MACEs in individuals diagnosed with type 2 diabetes mellitus (T2DM).

**Methods:**

In total, 4332 patients diagnosed with T2DM who underwent coronary angiography (CAG) were included and categorized into two groups (CHD and non-CHD) based on the CAG results. We used a correlation analysis to explore the potential links between the levels of circulating Lp(a) and CHD severity. Cox regression analysis was performed to evaluate MACEs.

**Results:**

The concentrations of circulating Lp(a) were markedly elevated in the CHD group and positively correlated with disease severity. Our results indicate that elevated circulating Lp(a) is a crucial risk factor that significantly contributes to both the progression and severity of CHD. The differences between the two groups are evident in the risk of CHD occurrence [odds ratio (OR) = 1.597, 95 % confidence interval (CI): 1.354–1.893, *p* < 0.001], the different levels of vessel involvement (OR = 1.908 for triple-vessel *vs*. single-vessel disease, 95 % CI: 1.401–2.711, *p* < 0.001), and their relation to the Gensini Score (OR = 2.002 for high *vs*. low GS, 95 % CI: 1.514–2.881, *p* < 0.001). Over the course of the 7-year follow-up period, the multivariate Cox regression analysis indicated that increased levels Lp(a) levels are independently associated with the occurrence of MACEs [hazard ratio (HR) = 1.915, 95 % CI: 1.571–2.493, *p* < 0.001].

**Conclusion:**

We confirmed a positive correlation among circulating Lp(a) levels, CHD lesions count, and Gensini scores. Moreover, Lp(a) levels have predictive significance for the occurrence of MACEs in T2DM patients.

## 1 Introduction

With economic development, the public health challenge posed by type 2 diabetes mellitus (T2DM) is increasing, as it remains one of the most rapidly increasing chronic diseases worldwide [[1], [2], [3]]. The global prevalence of diabetes is forecasted to escalate dramatically, rising from 536.6 million cases to a staggering 783.2 million by the year 2045 [4,5], with T2DM comprising 90–95 % of these cases [6]. Diabetes is commonly identified by elevated blood sugar levels, which arise due to either the insufficient secretion of insulin by pancreatic β-cells or the development of insulin resistance in individuals with T2DM. This condition elevates the risk of cardiovascular diseases, neuropathy, nephropathy, and retinopathy in affected individuals [7,8]. Diabetes and diabetes-related complications diminish patients’ quality of life and impose significant economic and social costs [9].

Lipoprotein(a), commonly referred to as Lp(a), is a variant closely resembling low-density lipoprotein cholesterol (LDL-C). Its primary structure is composed of an apolipoprotein B (apoB) molecule that is covalently bonded to an apolipoprotein (a) [apo(a)] molecule through a disulfide linkage, which is a pathological marker of Lp(a) [10]. The elevated levels of Lp(a) serve a significant function in promoting the formation and progression of arterial plaques, and therefore, it has long been considered a key factor in the early formation and later exacerbation of coronary heart disease (CHD) [11]. Recent research suggests that extremely low circulating Lp(a) levels may increase the risk of developing T2DM [12]. The variability in concentration, ranging from approximately 30–70 %, can be attributed to the significant polymorphism of Kringle-IV (K-IV) in the Lp(a) structure. The number of repeats within the K-IV structure of Lp(a) influences not only the size of apolipoprotein(a) [apo(a)] particles but also their concentration. Fewer K-IV repeats result in smaller, more pathogenic particles [13]. The size of Lp(a) particles can exceed 40, which is a unique phenomenon that differs from other circulating proteins that usually have a certain quality [14,15]. The *LPA* gene genetically influences both the levels and particle size of circulating Lp(a) in patients [16]; however, it has also been reported that non-genetic factors may also regulate the Lp(a) concentration [13]. Lp(a) promotes atherosclerosis through various non-redundant mechanisms, including oxidizing upon entering the blood vessel wall, forming foam cells, inducing an inflammatory response to plaques, and producing highly immunogenic and proinflammatory oxidized LDL-C [[17], [18], [19]].

Despite being considered a risk factor for atherosclerosis and associated conditions [13], Lp(a) is believed to have limited predictive value for cardiovascular events among patients with T2DM. Moreover, previous studies have mostly included Caucasian populations from Europe, leaving a gap in research regarding Lp(a) concentrations among Han Chinese individuals with T2DM. Therefore, this study aimed to systematically assess whether high levels of circulating Lp(a) are directly associated with the severity of CHD in individuals with T2DM, and to determine if Lp(a) independently predicts cardiovascular events over a long-term follow-up.

## 2 Material and methods

### 2.1 Study population

**Fig. 1:**
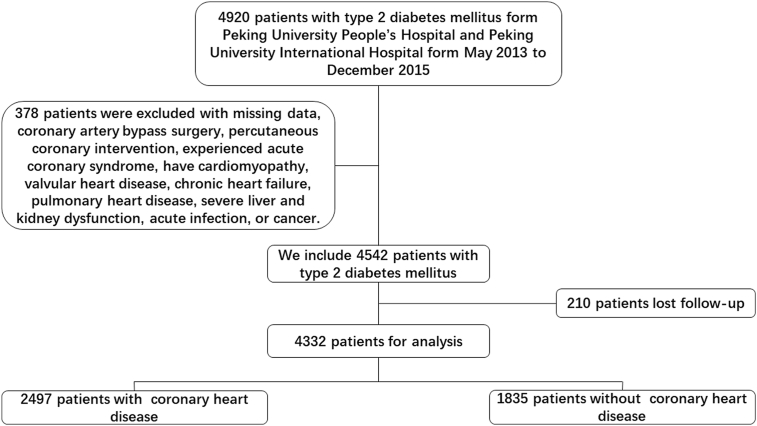
The flow chart of the patients' selection process.

Between May 2013 and December 2015, we enrolled 4332 patients with T2DM (1824 males and 2508 females) who received treatment at Peking University People's Hospital and Peking University International Hospital. Among these individuals, 2497 (1033 males and 1464 females) had been diagnosed with CHD (CHD group), and 1835 patients (791 males and 1044 females) had not (non-CHD group). Follow-ups were conducted by experienced nurses or physicians via in-person or telephone interviews every 6 months over a maximum duration of 7 years. Fig. 1 depicts the flowchart of the study design. Major adverse cardiovascular events (MACEs) were defined as cardiovascular mortality, hospitalization for unstable angina, non-fatal stroke, heart failure, and non-fatal myocardial infarction (MI) [20] (Supplementary Table 1).

The diagnosis of patients with T2DM was established based on the guidelines provided by the American Diabetes Association in 2012 [21]: 1) during the oral 75-g glucose tolerance test, the patient's fasting blood glucose (FBG) levels must be at least 126 mg/dL (7.0 mmol/L), or the levels of 2-h blood glucose (2-h BG) must be above 200 mg/dL (11.1 mmol/L); 2) glycated hemoglobin A1c (HbA_1c_) at above 6.5 %; or 3) patients exhibiting common typical diabetes symptoms and having a random blood glucose test result of 200 mg/dL (11.1 mmol/L) or higher.

Coronary angiography (CAG) results were used to diagnose CHD as exhibiting >50 % closure or stenosis of at least one main coronary artery, and the Gensini score (GS) is a detailed approach to assessing the severity of coronary artery obstruction in CHD. It involves measuring the extent of narrowing in the coronary arteries and taking into account the critical locations of these narrowings within the coronary system [22] (Supplementary Table 1).

Meanwhile, the following criteria were used to exclude participants [22]: 1) percutaneous coronary intervention with stent placement during the 3 months leading up to enrollment; 2) previous coronary artery bypass grafting surgery; 3) acute coronary syndrome <6 months prior to recruitment; 4) conditions such as cardiomyopathy, or persistent heart failure; 5) renal insufficiency; 6) cor pulmonale; 7) significant liver dysfunction; and 8) acute infection or cancer.

This study was approved by the Ethics Committee of Peking University People's Hospital (2018PHB155-01) and adhered to the ethical principle outlined in the Declaration of Helsinki guidelines. All participants were fully informed about the study's objectives, methods, possible risks, and anticipated benefits. They voluntarily participated in the study after providing written informed consent, ensuring their rights and well-being were safeguarded throughout the research.

### 2.2 Laboratory testing indicators

Patients’ blood samples were obtained following a minimum 12-h fast, centrifuged at 3500 rpm for 10 min, with the temperature kept between 10 and 15 °C, then stored in a −80 °C freezer. The circulating Lp(a) concentrations were quantified utilizing an advanced immunoturbidimetric assay kit from Roche (Roche Inc., Basel, Switzerland). The Beckman AU5800 chemistry analyzer system (Beckman Coulter Inc., California, USA) was used to measure FBG, homocysteine (HCY), a comprehensive lipid profile, and hypersensitive C-reactive protein (hs-CRP). The lipid profile encompasses concentrations of LDL-C, apoB, total cholesterol (TC), high-density lipoprotein cholesterol (HDL-C), Triglycerides (TG), apolipoprotein A-1 (apoA1), and small dense low-density lipoprotein cholesterol (sdLDL-C). HbA_1c_ concentrations were determined using the high-performance liquid chromatography system provided by Trinity Biotech (Trinity Biotech Inc., Bray, Ireland).

### 2.3 Statistical analyses

Each quantitative variable dataset must initially be subjected to a one-sample K-S test to assess its conformity to a normal distribution. For data following a normal distribution, group differences were evaluated using ANOVA or Student's t-test. The results are expressed as mean ± standard deviation. Continuous data that followed a non-normal distribution were evaluated for group differences using the Kruskal–Wallis test or the Mann–Whitney *U* test. Results are expressed as the median (inter-quartile range). The chi-square test was applied for comparing groups with percentage data. Spearman's correlation analysis was utilized to examine associations among the indicators. Logistic analyses assessed whether Lp(a) levels act as an independent predictor for CHD severity. The incidence of MACEs between groups was analyzed using the Kaplan–Meier test. Cox regression analysis was employed to investigate the relationship between circulating Lp(a) concentrations and the occurrence of MACEs. A *p*-value of <0.05 was considered statistically significant. Statistical analyses were conducted using IBM SPSS Statistics version 24 (IBM Inc., New York, USA) and GraphPad Prism version 8 (GraphPad Software Inc., California, USA).

## 3 Results

### 3.1 The baseline characteristics of the entire study cohort

Initially, we assessed the general profiles and clinical laboratory results of the participants. The specific outcome for the study cohort are presented in Table 1. No significant differences were observed in the following parameters: gender (male), body mass index (BMI), age, glycemic control, duration of diabetes, prevalence of hypertension, family history of diabetes, alcohol consumption, smoking prevalence, diabetic neuropathy, statin drug therapy, FBG, hs-CRP, TG, HBA_1c_, or HCY levels. Compared to the non-CHD group, the CHD group showed higher levels of family history of CHD, diabetic eye disease, statin drug therapy, serum sdLDL-C, LDL-C, apoB, TC and Lp(a) [41.70 (47.37) nmol/L *vs*. 37.97 (32.94) nmol/L, *p* < 0.001]. In contrast, HDL-C and apoA1 levels were markedly lower. The cohort was divided into three subgroups based on tertile levels of circulating Lp(a) to investigate the general and clinical characteristics of patients at different Lp(a) levels (Table 2). In the higher Lp(a) group, there were significantly elevated levels of diabetic eye disease, apoB, hs-CRP, TC, LDL-C, sdLDL-C, and HCY, compared to the lower Lp(a) group (*p* < 0.05). Conversely, the high Lp(a) group exhibited markedly lower levels of apoA1 and HDL-C (*p* < 0.05). No differences were found in age, BMI, gender (male), hypertension, smoking prevalence, alcohol consumption, glycemic control, duration of diabetes, diabetic neuropathy, statin drug therapy, family history of CHD or diabetes, FBG, HbA_1c_, or TG levels (*p* > 0.05).

**Table 1:** Baseline characteristics in type 2 diabetic patients.

	Total	CHD group	Non-CHD group	*p* value
Clinical characteristics				
N (%)	4332	2497 (57.64 %)	1835 (42.36 %)	–
Age (years)	55.13 ± 11.57	54.96 ± 10.79	55.53 ± 12.32	0.456
Male (%)	1824 (42.11 %)	1033 (41.37 %)	791 (43.11 %)	0.253
BMI (kg/m^2^)	24.68 ± 3.01	24.58 ± 2.90	24.85 ± 3.12	0.244
Hypertension (%)	3094 (71.42 %)	1810 (72.49 %)	1284 (69.97 %)	0.070
Smoking (%)	1249 (28.83 %)	696 (27.87 %)	553 (30.14 %)	0.109
Consumers of alcohol (%)	1362 (31.44 %)	804 (32.20 %)	558 (30.41 %)	0.210
Family history of CHD (%)	1271 (29.34 %)	772 (30.92 %)	499 (27.19 %)	0.008
Family history of MD (%)	1874 (43.26 %)	1063 (42.57 %)	811 (44.20 %)	0.286
Blood sugar is well controlled (%)	2980 (68.79 %)	1697 (67.96 %)	1283 (69.91 %)	0.174
Duration of diabetes >5 years (%)	932 (21.51 %)	551 (22.06 %)	381 (20.76 %)	0.312
Diabetic eye disease (%)	789 (18.21 %)	498 (19.94 %)	291 (15.85 %)	0.001
Diabetic neuropathy (%)	1231 (28.42 %)	723 (28.95 %)	508 (27.68 %)	0.375
Statin drug therapy (%)	2821(65.12 %)	2298 (92.03 %)	523 (28.50 %)	<0.001
Laboratory variables				
FBG (mmol/L)	7.49 ± 1.15	7.53 ± 1.19	7.41 ± 1.07	0.121
HbA_1c_ (%)	7.3 ± 1.1	7.3 ± 1.1	7.2 ± 1.0	0.513
ApoB (mg/dL)	84.60 (28.23)	88.31 (37.70)	84.17 (21.6)	0.005
ApoA1 (mg/dL)	139.80 (38.55)	138.70 (42.80)	141.00 (33.10)	0.002
Total cholesterol (mmol/L)	4.43 (1.32)	4.47 (1.71)	4.39 (0.99)	0.009
Triglycerides (mmol/L)	1.32 (0.87)	1.31 (0.83)	1.33 (0.95)	0.390
HDL-C (mmol/L)	1.08 (0.32)	1.05 (0.29)	1.11 (0.40)	0.006
LDL-C (mmol/L)	2.72 (0.95)	2.78 (1.35)	2.69 (0.63)	0.010
hs-CRP (mg/L)	1.40 (1.68)	1.60 (1.75)	1.22 (1.58)	0.249
HCY (umol/L)	10.86 (6.86)	10.90 (8.22)	10.68 (5.44)	0.247
sdLDL-C (mmol/L)	0.76 (0.38)	0.78 (0.48)	0.72 (0.30)	0.002
Lp(a) (nmol/L)	40.51 (39.68)	41.70 (47.37)	37.97 (32.94)	<0.001

Data are reported as means ± SD or n (%), median (interquartile ranges). SD: Standard deviation.

BMI: body mass index; CHD: coronary heart disease; DM: diabetes mellitus; FPG: fasting plasma glucose; HbA1c: Hemoglobin A1c; apoB: apolipoprotein B; apoA1: apolipoprotein A1; HDL-C: high density lipoprotein cholesterol; LDL-C: low density lipoprotein cholesterol; Hs-CRP: hypersensitive C-reactive protein; HCY: homocysteine; sdLDL-C: small dense low-density lipoprotein cholesterol; Lp(a): lipoprotein (a).

Statistical analysis was performed with the student's *t*-test or Mann-Whitney *U* test and with Chi-square test for categorical variables.

**Table 2:** Baseline characteristics of patients with type 2 diabetes at different Lp(a) levels.

Variables	Lp(a) concentration (nmol/L)			*p* value
	Low Lp(a)	Mid Lp(a)	High Lp(a)	
	<31.51	31.51–53.58	>53.58	
Clinical characteristics				
N (%)	1443 (33.31 %)	1447 (33.40 %)	1442 (33.29 %)	–
Age (years)	54.69 ± 11.68	55.50 ± 11.29	55.37 ± 11.30	0.556
Male (%)	579 (40.12 %)	616 (42.57 %)	629 (43.62 %)	0.149
BMI (kg/m^2^)	24.66 ± 3.14	24.58 ± 3.08	24.81 ± 2.98	0.778
Hypertension (%)	1006 (69.72 %)	1029 (71.11 %)	1059 (73.44 %)	0.082
Smoking (%)	426 (29.52 %)	390 (26.95 %)	433 (30.03 %)	0.147
Consumers of alcohol (%)	457 (31.67 %)	441 (30.48 %)	464 (32.18 %)	0.600
Family history of CHD (%)	397 (27.51 %)	423 (29.23 %)	451 (31.28 %)	0.085
Family history of MD (%)	636 (44.07 %)	615 (42.50 %)	623 (43.20 %)	0.694
Blood sugar is well controlled (%)	1018 (70.55 %)	991 (68.48 %)	971 (67.34 %)	0.169
Duration of diabetes >5 years (%)	329 (22.80 %)	302 (20.87 %)	301 (20.87 %)	0.347
Diabetic eye disease (%)	235 (16.29 %)	268 (18.52 %)	286 (19.83 %)^a^	0.044
Diabetic neuropathy (%)	395 (27.37 %)	421 (29.09 %)	415 (28.78 %)	0.551
Statin drug therapy (%)	915 (63.41 %)	971 (67.10 %)	935 (64.84 %)	0.110
Laboratory variables				
FPG (mmol/L)	7.45 ± 1.47	7.55 ± 1.26	7.51 ± 1.13	0.222
HbA_1c_ (%)	7.3 ± 1.4	7.3 ± 1.1	7.2 ± 1.1	0.915
ApoB (mg/dL)	78.60 (27.34)	83.70 (27.52)^a^	91.14 (31.87)^ab^	<0.001
ApoA1 (mg/dL)	142.60 (38.75)	140.45 (38.82)	136.50 (35.42)^ab^	0.006
Total cholesterol (mmol/L)	4.29 (1.38)	4.40 (1.24)	4.60 (1.26)^ab^	<0.001
Triglycerides (mmol/L)	1.27 (0.83)	1.37 (0.93)	1.34 (0.90)	0.646
HDL-C (mmol/L)	1.11 (0.36)	1.08 (0.31)	1.05 (0.29)^a^	0.007
LDL-C (mmol/L)	2.56 (0.94)	2.67 (0.90)	2.88 (0.98)^ab^	<0.001
hs-CRP (mg/L)	1.26 (1.55)	1.27 (1.73)	1.63 (1.77)^ab^	0.024
HCY (umol/L)	10.12 (5.56)	10.97 (6.53)^a^	11.41 (9.31)^a^	0.001
sdLDL-C (mmol/L)	0.68 (0.40)	0.74 (0.30)^a^	0.79 (0.45)^ab^	<0.001
Lp(a) (nmol/L)	22.38 (7.28)	40.50 (10.24)^a^	84.05 (53.92)^ab^	–

Data are reported as means ± SD or n(%), median (interquartile ranges). SD: Standard deviation.

BMI: body mass index; CHD: coronary heart disease; DM: diabetes mellitus; FPG: fasting plasma glucose; HbA1c: Hemoglobin A1c; apoB: apolipoprotein B; apoA1: apolipoprotein A1; HDL-C: high density lipoprotein cholesterol; LDL-C: low density lipoprotein cholesterol; Lp(a): lipoprotein (a); Hs-CRP: hypersensitive C-reactive protein; HCY: homocysteine; sdLDL-C: small dense low-density lipoprotein cholesterol; Lp(a): lipoprotein (a).

Statistical analysis was performed with the ANOVA or Kruskal – Wall test and with Chi-square test for categorical variables.

^a^: Shows that the p < 0.05 compared with the Low Lp(a) group.

^b^: Shows that the p < 0.05 compared with the Mid Lp(a) group.

### 3.2 The severity of coronary artery obstruction and circulating Lp(a) levels

**Fig. 2:**
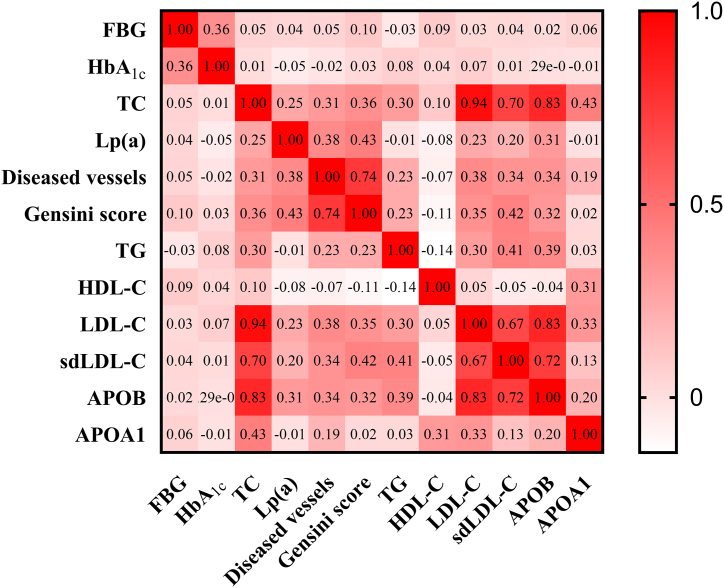
Correlation between Lp(a) and severity of coronary heart disease obstruction (diseased vessels: r = 0.38, *p* < 0.001; Gensini score: r = 0.43, *p* < 0.001).

We classified CHD patients into three distinct groups based on their circulating Lp(a) levels to investigate more thoroughly the relationship between varying Lp(a) concentrations and the degree of CHD obstruction. The analysis showed that patients with higher Lp(a) concentrations of exhibited a significantly increased incidence of triple-vessel disease and higher Gensini Scores [22.62 % *vs*. 44.00 % *vs*. 50.84 %; 26 (22) *vs*. 33 (23) *vs*. 39 (23), *p* < 0.001] compared to those with lower Lp(a) levels. Conversely, the prevalence of single-vessel disease was reduced in the high Lp(a) group (47.65 % *vs*. 21.70 % *vs*. 19.95 %, *p* < 0.001) (Supplementary Table 2). Furthermore, there was a significant positive correlation between serum Lp(a) levels and both the number of diseased vessels and the Gensini Score (Spearman's correlation analysis, r = 0.38 and 0.43, *p* < 0.001) (Fig. 2). These finding suggest that elevated Lp(a) concentrations may exacerbate the severity of coronary artery disease.

### 3.3 Serum Lp(a) concentrations and CHD risk in individuals with T2DM

**Fig. 3:**
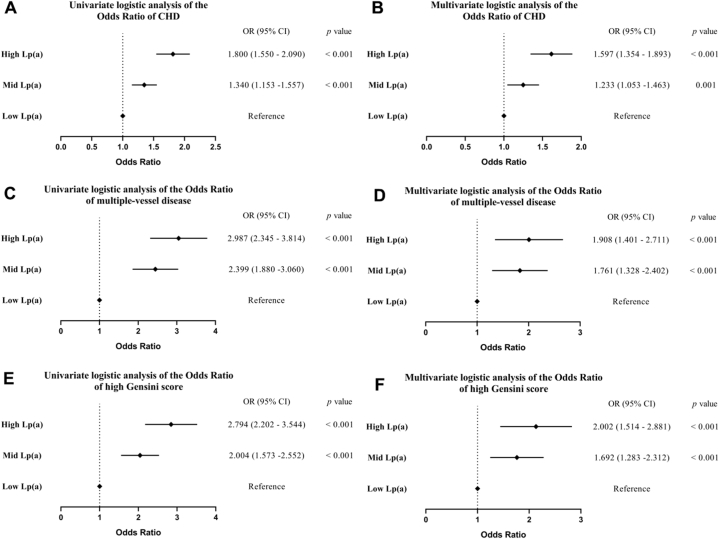
Logistic regression analysis between tertile of circulating Lp(a) level and CHD, multiple-vessel disease and high Gensini score. A. univariate logistic regression analysis between circulating Lp(a) and CHD; B. multivariate logistic regression analysis between circulating Lp(a) and CHD; C. univariate logistic regression analysis between circulating Lp(a) and multiple-vessel disease; D. multivariate logistic regression analysis between circulating Lp(a) and multiple-vessel disease; E. univariate logistic regression analysis between circulating Lp(a) and Gensini score; F. multivariate logistic regression analysis between circulating Lp(a) and Gensini score.

To explore the relationship between Lp(a) levels and CHD severity, we categorized CHD patients based on the number of diseased vessels—single-, double-, and triple-vessel and GS categories—low, mid, and high derived from CAG results. Furthermore, patients were stratified into tertiles according to their Lp(a) concentrations. The low Lp(a) tertile served as the reference group for assessing the risk and severity of arterial obstruction in the middle and high Lp(a) tertiles. We employed binary multivariate logistic regression models to derive these insights. The results of univariate logistic regression analysis revealed a positive correlation between circulating Lp(a) levels and the OR for both the risk and severity of CHD. Notably, individuals in the highest Lp(a) tertile demonstrated a significantly elevated risk of CHD compared to those in the lowest tertile (OR = 1.800, 95 % CI: 1.550–2.090, *p* < 0.001). Moreover, the odds of developing triple-vessel disease were higher than those for single vessel disease (OR = 2.987, 95 % CI: 2.345–3.814, *p* < 0.001). Additionally, GS values were also higher in these groups. (OR = 2.794, 95 % CI: 2.202–3.544, *p* < 0.001) (Fig. 3 A, C, E). In further analyses, we adjusted for factors such as age, BMI, sex, alcohol consumption, hypertension, smoking, glycemic control, duration of diabetes, diabetic complication, statin therapy, family history of CHD and diabetes, and various biochemical markers. The trends persisted, with higher odds consistently observed in the higher tertiles (OR = 1.597 for CHD *vs.* non-CHD, 95 % CI: 1.354–1.897, *p* < 0.001; OR = 1.908 for triple-vessel *vs.* single-vessel disease, 95 % CI: 1.401–2.711, *p* < 0.001; OR = 2.002 for high GS *vs.* low GS, 95 % CI: 1.514–2.881, *p* < 0.001) (Fig. 3 B, D, F). These findings underscore that higher Lp(a) levels are associated with more severe coronary artery disease.

### 3.4 Circulating Lp(a) levels and MACEs outcomes

**Fig. 4:**
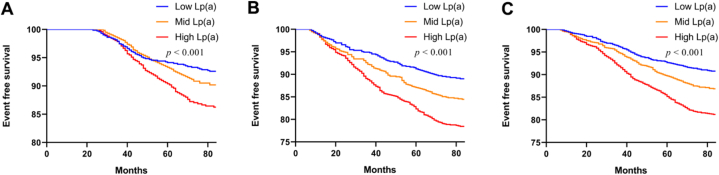
Kaplan-Meier curve based on tertile of circulating Lp(a) level among different patients. A. in the non-CHD group; B. in the CHD group; C. in all participants.

In total, 594 (13.71 %) patients experienced MACEs during the 7 years follow-up period: 411 (16.46 %) patients from the CHD group and 183 (9,97 %) patients from a subset of the non-CHD group who developed CHD during the follow-up period (n = 352; 19.18 %). Patients who experienced MACEs had a greater prevalence of CHD family history and significantly higher levels of apoB, TC, sdLDL-C, and Lp(a) [46.11 (41.18) nmol/L *vs*. 39.43 (39.87) nmol/L, *p* = 0.001] compared to patients who did not experience MACEs (Table 3). We classified all participants into three distinct groups based on tertiles of circulating Lp(a) levels (low, medium, high) to investigate the relationship between varying Lp(a) levels and MACEs. The analysis indicates a notably higher incidence of MACEs in individuals with higher Lp(a) levels compared to those with lower levels, a trend observed consistently across different population groups: non-CHD group (7.41 % *vs* 9.82 % *vs* 13.75 %, *p* < 0.001) (Supplementary Table 3), the CHD group (10.99 % *vs*. 15.55 % *vs*. 21.54 %, *p* < 0.001) (Supplementary Table 4), and the overall cohort (9.22 % *vs* 13.13 % *vs*. 18.79 %, *p* < 0.001) (Supplementary Table 5). The Kaplan-Meier analysis demonstrated that elevated circulating Lp(a) levels were linked to a more frequent occurrence of MACEs among all patients (Fig. 4. C). Comparable outcomes were noted in the remaining two groups (Fig. 4 A, B).

**Table 3:** Baseline characteristics in all subjects with or without MACEs.

	Total	MACEs group	Non- MACEs group	*p* value
Clinical characteristics				
N (%)	4332	594 (13.71 %)	3738 (86.29 %)	–
Age (years)	55.13 ± 11.57	56.05 ± 11.83	54.99 ± 11.36	0.339
Male (%)	1824 (42.11 %)	261 (43.94 %)	1563 (41.81 %)	0.330
BMI (kg/m^2^)	24.68 ± 3.01	25.21 ± 2.90	24.60 ± 3.09	0.123
Hypertension (%)	3094 (71.42 %)	436 (73.40 %)	2658 (71.11 %)	0.251
Smoking (%)	1249 (28.83 %)	165 (27.78 %)	1084 (29.00 %)	0.541
Consumers of alcohol (%)	1362 (31.44 %)	198 (33.33 %)	1164 (31.14 %)	0.285
Family history of CHD (%)	1271 (29.34 %)	201 (33.83 %)	1070 (28.62 %)	0.010
Family history of MD (%)	1874 (43.26 %)	263 (44.27 %)	1611 (43.10 %)	0.590
Blood sugar is well controlled (%)	2980 (68.79 %)	410 (69.02 %)	2570 (68.75 %)	0.924
Duration of diabetes >5 years (%)	932 (21.51 %)	136 (22.90 %)	796 (21.29 %)	0.390
Diabetic eye disease (%)	789 (18.21 %)	104 (17.51 %)	685 (18.33 %)	0.689
Diabetic neuropathy (%)	1231 (28.42 %)	155 (26.09 %)	1076 (28.79 %)	0.186
Statin drug therapy (%)	2821(65.12 %)	405 (68.18 %)	2416 (64.63 %)	0.095
Laboratory variables				
FPG (mmol/L)	7.49 ± 1.15	7.63 ± 1.26	7.46 ± 1.13	0.124
HbA_1c_ (%)	7.3 ± 1.1	7.3 ± 1.1	7.2 ± 1.1	0.414
ApoB (mg/dL)	84.60 (28.23)	88.92 (35.76)	83.88 (27.84)	0.028
ApoA1 (mg/dL)	139.80 (38.55)	141.45 (37.60)	139.60 (38.40)	0.234
Total cholesterol (mmol/L)	4.43 (1.32)	4.59 (1.30)	4.42 (1.33)	0.013
Triglycerides (mmol/L)	1.32 (0.87)	1.37 (0.91)	1.30 (0.86)	0.402
HDL-C (mmol/L)	1.08 (0.32)	1.07 (0.30)	1.08 (0.32)	0.632
LDL-C (mmol/L)	2.72 (0.95)	2.72 (1.16)	2.72 (0.92)	0.157
hs-CRP (mg/L)	1.40 (1.68)	1.55 (1.63)	1.35 (1.69)	0.406
HCY (umol/L)	10.86 (6.86)	10.94 (6.05)	10.78 (6.95)	0.810
sdLDL-C (mmol/L)	0.76 (0.38)	0.83 (0.43)	0.74 (0.36)	0.013
Lp(a) (nmol/L)	40.51 (39.68)	46.11 (41.18)	39.43 (39.87)	0.001

Data are reported as means ± SD or n(%), median (interquartile ranges). SD: Standard deviation.

MACEs: major cardiovascular events; BMI: body mass index; CHD: coronary heart disease; DM: diabetes mellitus; FPG: fasting plasma glucose; HbA1c: Hemoglobin A1c; apoB: apolipoprotein B; apoA1: apolipoprotein A1; HDL-C: high density lipoprotein cholesterol; LDL-C: low density lipoprotein cholesterol; HsCRP: hypersensitive C-reactive protein; HCY: homocysteine; sdLDL-C: small dense low-density lipoprotein cholesterol; Lp(a): lipoprotein (a).

Statistical analysis was performed with the student's *t*-test or Mann-Whitney *U* test and with Chi-square test for categorical variables.

**Fig. 5:**
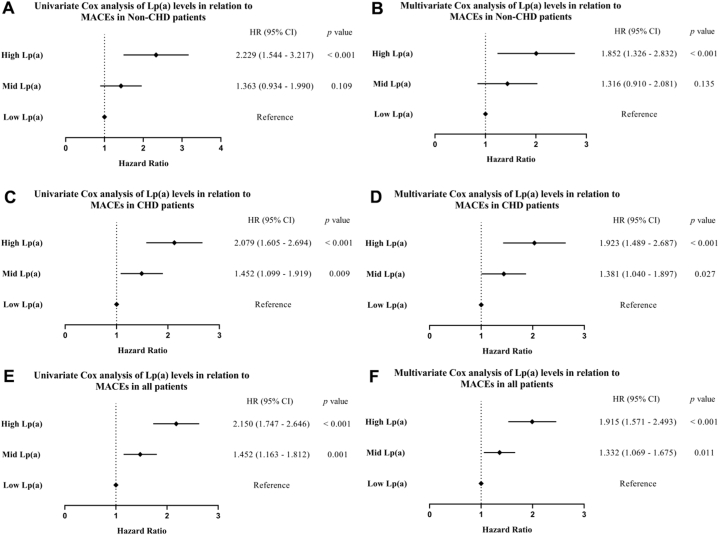
Cox regression analysis of tertile of circulating Lp(a) level in different patients. A. univariate Cox regression analysis in non-CHD group; B. multivariate Cox regression analysis in non-CHD group; C. univariate Cox regression analysis in CHD group; D. multivariate Cox regression analysis in CHD group; E. univariate Cox regression analysis in all participants; F. multivariate Cox regression analysis in all participants.

Univariate Cox regression analysis of the three groups revealed that, compared to the group with low Lp(a) levels (reference group), the group with higher Lp(a) levels exhibited a significantly increased risk of MACEs in the non-CHD group, CHD group, and the entire cohort. The hazard ratios were HR = 2.229 (95%CI: 1.544–3.217, *p* < 0.001), HR = 2.079 (95%CI: 1.605–2.694, *p* < 0.001), and HR = 2.150 (95%CI: 1.747–2.646, *p* < 0.001) respectively (Fig. 5 A, C, E). After adjusting for confounding factors, the results remained consistent. The adjusted hazard ratios (HR) for the CHD group, the non-CHD group, and the entire cohort were 1.852, 1.923, and 1.915, respectively (all *p* < 0.001) (Fig. 5 B, D, F). These findings indicate that elevated circulating Lp(a) levels are associated with an increased risk of future MACEs.

## 4 Discussion

In the past few decades, various studies, including epidemiological studies, randomized controlled trials, meta-analyses, and metabolomics and proteomics studies, have confirmed that high circulating Lp(a) levels contribute to myocardial infarction, peripheral arterial disease, and stroke [[23], [24], [25], [26]]. Moreover, research on individuals without prior cardiovascular disease has demonstrated that increased levels of circulating Lp(a) somewhat elevate the risk of cerebrovascular and cardiovascular diseases [27,28]. Among 20793 CHD cases and 27540 controls from the CHD ExomePlus Consortium, 43 independent variants with genome-wide significance for CHD susceptibility were identified, with the most effective variants related to lipid metabolism and inflammation [29,30]. In our current state of knowledge, research on the association between the levels of circulating Lp(a) and the prediction of cardiovascular and cerebrovascular outcomes in Northern Chinese Han patients with T2DM is currently limited. Our research is the first comprehensive longitudinal investigation. Our research findings indicate that increased circulating Lp(a) levels could potentially heighten the likelihood of developing atherosclerotic heart disease. Over our 7-year follow-up observation, we observed that, in patients with T2DM, the probability of experiencing MACEs in the future increased with higher circulating levels of Lp(a).

Lp(a) is thought to be positively correlated with the incidence of cardiovascular events due to its unique structural properties. Besides promoting atherosclerosis, Lp(a) interferes with fibrinolysis and encourages thrombosis [31]. Pathologically, Lp(a) binds to arterial intimal glycosaminoglycans with a 4-fold higher affinity for LDL-C [32]. Therefore, atherosclerotic plaques harbor Lp(a)-glycosaminoglycan complexes that draw in immunogenic cells, such as monocytes and macrophages, triggering inflammatory processes [33]. Moreover, Lp(a) is involved in the oxidation of cell-surface phospholipids by free radicals. Modified Lp(a) binds to glycosaminoglycan in blood vessels, initiating inflammatory processes, releasing cytokine and interleukin, and ultimately plaque formation [34]. Similarly, Lp(a) has been proposed to promote clotting after plaque rupture, thereby increasing the occurrence of cardiovascular events. This is because apo(a) contains a large amount of phosphoproteins, and Lp(a) binds strongly to the lysine group of fibrin [35]. Moreover, the apo(a) complex has been shown to have partial resistance to cleavage by fibrinolytic enzymes. Apo(a) hinders the tissue plasminogen activator enzyme from converting plasminogen into plasmin [36,37]. Furthermore, elevated circulating Lp(a) levels can diminish HDL-C's protective role against arterial narrowing, thereby hastening the progression of CHD [38]. Our research results have confirmed this.

To date, research on the link between diabetes and Lp(a) has yielded mixed results. The European Atherosclerosis Society's latest consensus statement identifies very low circulating Lp(a) levels as a potential cause of diabetes [13]. However, evidence suggests that reducing the Lp(a) concentration of the highest quintile to the median value could potentially reduce the risk of CHD without increasing the risk of T2DM [12]. Littmann and colleagues discovered that diabetic patients who maintained good metabolic control tended to have lower Lp(a) levels. Additionally, they also identified a notable positive correlation between HbA_1c_ and the levels of Lp(a), indicating that circulating Lp(a) may impact insulin synthesis and secretion within cells [23]. Conversely, Dai et al. [39] observed no differences in FBG and HbA1_c_ levels among populations with varying Lp(a) levels in Japan. Our findings align with theirs. This discrepancy in results may be because prospective studies can improve the management of blood glucose control in diabetic patients by enhancing insulin treatment, whereas cross-sectional studies cannot.

Similarly, whether high circulating Lp(a) levels will increase the incidence of cardiovascular events in diabetic patients in the future remains a subject of controversy. For example, O'Donoghue et al. reported that elevated levels of circulating Lp(a) heighten the likelihood of patients developing cardiovascular disease, regardless of LDL-C levels [40]. However, a multiethnic study did not replicate these results in the majority of Caucasian populations [41]. This was primarily atrributed to the notable variations in circulating Lp(a) levels across different populations, especially because the study population was mostly from Africa. Our novel study on T2DM in northern Chinese Han patients supports the research by O'Donoghue et al. and provides complementary data to support the finding that future cardiovascular event risk in T2DM patients may be heightened by elevated circulating Lp(a) levels. Furthermore, it shows that Lp(a) levels are directly linked to the degree of CHD obstruction. In clinical practice, besides focusing on traditional lipid markers like LDL-C, it is important to also consider circulating Lp(a) levels.

## 5 Limitations

There were several limitations impacting this study. Firstly, the participation of only two centers in northern China could have introduced potential selection bias, necessitating further validation of our findings via broader, multicenter studies. Secondly, the exclusion of healthy controls and prediabetic patients from our study cohort limits our capacity to directly associate lower Lp(a) levels with diabetes. Consequently, future research efforts focusing on these specific populations are deemed essential.

## 6 Conclusion

According to our study, the levels of circulating Lp(a) are positively associated with multivessel disease and GS in patients with T2DM and CHD. Moreover, our findings highlight the significance of elevated Lp(a) levels as a standalone predictor for CHD and MACEs in T2DM patients in China. Therefore, clinicians should consider Lp(a) in conjunction with LDL-C when evaluating CHD.

## Funding sources

This study was supported by the 10.13039/501100005089Beijing Natural Science Foundation (7222194), the 10.13039/501100004735Natural Science Foundation of Hunan Province (2021JJ41023) and the Beijing Major Epidemic Prevention and Control Key Specialty Project-Medical Laboratory Excellence Project (2022).

## Data availability statement

Data will be made available on request.

## Consent for publication

Not applicable.

## CRediT authorship contribution statement

**Jun-Xu Gu:** Writing – review & editing, Writing – original draft, Data curation, Conceptualization. **Juan Huang:** Writing – original draft, Methodology, Data curation, Conceptualization. **Kun Wang:** Writing – original draft, Software, Conceptualization. **Yue Yin:** Methodology, Funding acquisition, Data curation. **Jun-Ling Fang:** Resources, Methodology. **Ai-Min Zhang:** Validation, Supervision, Software. **Shan-Shan Li:** Visualization, Software, Resources. **Xiao-Qin Yao:** Validation, Project administration. **Ming Yang:** Supervision, Software, Resources. **Na Zhang:** Validation, Investigation. **Mei Jia:** Writing – review & editing, Writing – original draft, Software, Resources, Data curation, Conceptualization. **Ming Su:** Writing – review & editing, Supervision, Resources, Formal analysis, Data curation.

## Declaration of competing interest

The authors declare that they have no known competing financial interests or personal relationships that could have appeared to influence the work reported in this paper.

## References

[1] ElSayed N.A., Aleppo G., Aroda V.R., Bannuru R.R., Brown F.M., Bruemmer D., Collins B.S., Hilliard M.E., Isaacs D., Johnson E.L. (2023). 2. Classification and diagnosis of diabetes: standards of care in diabetes-2023. Diabetes Care.

[2] Koziel K., Urbanska E.M. (2023). Kynurenine pathway in diabetes mellitus-novel pharmacological target?. Cells.

[3] Bloomgarden Z. (2023). A diabetes update. J. Diabetes.

[4] Zhang L., Zhang Y., Shen S., Wang X., Dong L., Li Q., Ren W., Li Y., Bai J., Gong Q. (2023). Safety and effectiveness of metformin plus lifestyle intervention compared with lifestyle intervention alone in preventing progression to diabetes in a Chinese population with impaired glucose regulation: a multicentre, open-label, randomised controlled trial. Lancet Diabetes Endocrinol..

[5] Sun H., Saeedi P., Karuranga S., Pinkepank M., Ogurtsova K., Duncan B.B., Stein C., Basit A., Chan J.C.N., Mbanya J.C. (2022). IDF Diabetes Atlas: global, regional and country-level diabetes prevalence estimates for 2021 and projections for 2045. Diabetes Res. Clin. Pract..

[6] Huang K., Liang Y., Wang K., Wu J., Luo H., Yi B. (2022). Influence of circulating nesfatin-1, GSH and SOD on insulin secretion in the development of T2DM. Front. Public Health.

[7] Huang K., Liang Y., Ma Y., Wu J., Luo H., Yi B. (2022). The variation and correlation of serum adiponectin, nesfatin-1, IL-6, and TNF-alpha levels in prediabetes. Front. Endocrinol..

[8] Lamina C., Ward N.C. (2022). Lipoprotein (a) and diabetes mellitus. Atherosclerosis.

[9] Schwartz G.G., Szarek M., Bittner V.A., Bhatt D.L., Diaz R., Goodman S.G., Jukema J.W., Loy M., Manvelian G., Pordy R. (2021). Relation of lipoprotein(a) levels to incident type 2 diabetes and modification by alirocumab treatment. Diabetes Care.

[10] Singh S.S., van der Toorn J.E., Sijbrands E.J.G., de Rijke Y.B., Kavousi M., Bos D. (2023). Lipoprotein(a) is associated with a larger systemic burden of arterial calcification. Eur Heart J Cardiovasc Imaging.

[11] Reyes-Soffer G., Ginsberg H.N., Berglund L., Duell P.B., Heffron S.P., Kamstrup P.R., Lloyd-Jones D.M., Marcovina S.M., Yeang C., Koschinsky M.L. (2022). Lipoprotein(a): a genetically determined, causal, and prevalent risk factor for atherosclerotic cardiovascular disease: a scientific statement from the American heart association. Arterioscler. Thromb. Vasc. Biol..

[12] Gudbjartsson D.F., Thorgeirsson G., Sulem P., Helgadottir A., Gylfason A., Saemundsdottir J., Bjornsson E., Norddahl G.L., Jonasdottir A., Jonasdottir A. (2019). Lipoprotein(a) concentration and risks of cardiovascular disease and diabetes. J. Am. Coll. Cardiol..

[13] Kronenberg F., Mora S., Stroes E.S.G., Ference B.A., Arsenault B.J., Berglund L., Dweck M.R., Koschinsky M., Lambert G., Mach F. (2022). Lipoprotein(a) in atherosclerotic cardiovascular disease and aortic stenosis: a European Atherosclerosis Society consensus statement. Eur. Heart J..

[14] Clarke R., Peden J.F., Hopewell J.C., Kyriakou T., Goel A., Heath S.C., Parish S., Barlera S., Franzosi M.G., Rust S. (2009). Genetic variants associated with Lp(a) lipoprotein level and coronary disease. N. Engl. J. Med..

[15] Law H.G., Khan M.A., Zhang W., Bang H., Rood J., Most M., Lefevre M., Berglund L., Enkhmaa B. (2023). Reducing saturated fat intake lowers LDL-C but increases Lp(a) levels in African Americans: the GET-READI feeding trial. J. Lipid Res..

[16] Tasdighi E., Adhikari R., Almaadawy O., Leucker T.M., Blaha M.J. (2023). LP(a): structure, genetics, associated cardiovascular risk, and emerging therapeutics. Annu. Rev. Pharmacol. Toxicol..

[17] Averna M.R., Cefalu A.B. (2023). Lp(a): a genetic cause of clinical FH in children. Eur. Heart J..

[18] Al Hageh C., Chacar S., Ghassibe-Sabbagh M., Platt D.E., Henschel A., Hamdan H., Gauguier D., El Murr Y., Alefishat E., Chammas E. (2023). Elevated Lp(a) levels correlate with severe and multiple coronary artery stenotic lesions. Vasc. Health Risk Manag..

[19] Raitakari O., Kartiosuo N., Pahkala K., Hutri-Kahonen N., Bazzano L.A., Chen W., Urbina E.M., Jacobs D.R., Sinaiko A., Steinberger J. (2023). Lipoprotein(a) in youth and prediction of major cardiovascular outcomes in adulthood. Circulation.

[20] Ray K.K., Raal F.J., Kallend D.G., Jaros M.J., Koenig W., Leiter L.A., Landmesser U., Schwartz G.G., Lawrence D., Friedman A. (2023). Inclisiran and cardiovascular events: a patient-level analysis of phase III trials. Eur. Heart J..

[21] American Diabetes A. (2012). Diagnosis and classification of diabetes mellitus. Diabetes Care.

[22] Huang J., Gu J.X., Wang K., Zhang A.M., Hong T.T., Li S.S., Yao X.Q., Yang M., Yin Y., Zhang N. (2023). Association between serum PCSK9 and coronary heart disease in patients with type 2 diabetes mellitus. Diabetol Metab Syndr.

[23] Littmann K., Wodaje T., Alvarsson M., Bottai M., Eriksson M., Parini P., Brinck J. (2020). The association of lipoprotein(a) plasma levels with prevalence of cardiovascular disease and metabolic control status in patients with type 1 diabetes. Diabetes Care.

[24] Mohammadi-Shemirani P., Chong M., Narula S., Perrot N., Conen D., Roberts J.D., Theriault S., Bosse Y., Lanktree M.B., Pigeyre M., Pare G. (2022). Elevated lipoprotein(a) and risk of atrial fibrillation: an observational and mendelian randomization study. J. Am. Coll. Cardiol..

[25] Willeit P., Ridker P.M., Nestel P.J., Simes J., Tonkin A.M., Pedersen T.R., Schwartz G.G., Olsson A.G., Colhoun H.M., Kronenberg F. (2018). Baseline and on-statin treatment lipoprotein(a) levels for prediction of cardiovascular events: individual patient-data meta-analysis of statin outcome trials. Lancet.

[26] Di Fusco S.A., Maggioni A.P., Scicchitano P., Zuin M., D'Elia E., Colivicchi F. (2023). Lipoprotein (a), inflammation, and atherosclerosis. J. Clin. Med..

[27] Gu J.X., Huang J., Li S.S., Zhou L.H., Yang M., Li Y., Zhang A.M., Yin Y., Zhang N., Jia M., Su M. (2022). Elevated lipoprotein(a) and genetic polymorphisms in the LPA gene may predict cardiovascular events. Sci. Rep..

[28] Suran D., Zavrsnik T., Kokol P., Kokol M., Sinkovic A., Naji F., Zavrsnik J., Blazun Vosner H., Kanic V. (2023). Lipoprotein(a) as a risk factor in a cohort of hospitalised cardiovascular patients: a retrospective clinical routine data analysis. J. Clin. Med..

[29] Lampsas S., Xenou M., Oikonomou E., Pantelidis P., Lysandrou A., Sarantos S., Goliopoulou A., Kalogeras K., Tsigkou V., Kalpis A. (2023). Lipoprotein(a) in atherosclerotic diseases: from pathophysiology to diagnosis and treatment. Molecules.

[30] Burgess S., Ference B.A., Staley J.R., Freitag D.F., Mason A.M., Nielsen S.F., Willeit P., Young R., Surendran P., Karthikeyan S. (2018). Association of LPA variants with risk of coronary disease and the implications for lipoprotein(a)-lowering therapies: a Mendelian randomization analysis. JAMA Cardiol.

[31] Zhang J., Jia L., Yang Y., Xiao A., Lin X. (2023). Lipoprotein (a) and myocardial infarction: impact on long-term mortality. Lipids Health Dis..

[32] Yi C., Junyi G., Fengju L., Qing Z., Jie C. (2023). Association between lipoprotein(a) and peripheral arterial disease in coronary artery bypass grafting patients. Clin. Cardiol..

[33] Kamstrup P.R. (2021). Lipoprotein(a) and cardiovascular disease. Clin. Chem..

[34] Zhang J., Liu M., Ferdous M., Zhao P., Li X. (2023). Serum lipoprotein(a) predicts 1-year major cardiovascular events in patients after percutaneous coronary intervention. Am J Transl Res.

[35] Boffa M.B. (2022). Beyond fibrinolysis: the confounding role of Lp(a) in thrombosis. Atherosclerosis.

[36] Kang C., Dominguez M., Loyau S., Miyata T., Durlach V., Angles-Cano E. (2002). Lp(a) particles mold fibrin-binding properties of apo(a) in size-dependent manner: a study with different-length recombinant apo(a), native Lp(a), and monoclonal antibody. Arterioscler. Thromb. Vasc. Biol..

[37] Siudut J., Natorska J., Wypasek E., Wiewiorka L., Ostrowska-Kaim E., Wisniowska-Smialek S., Plens K., Musialek P., Legutko J., Undas A. (2022). Apolipoproteins and lipoprotein(a) as factors modulating fibrin clot properties in patients with severe aortic stenosis. Atherosclerosis.

[38] Burdeynaya A.L., Afanasieva O.I., Ezhov M.V., Klesareva E.A., Saidova M.A., Pokrovsky S.N. (2023). Lipoprotein(a) and its autoantibodies in association with calcific aortic valve stenosis. Diseases.

[39] Dai K., Shiode N., Yoshii K., Kimura Y., Matsuo K., Jyuri Y., Tomomori S., Higaki T., Oi K., Kawase T. (2023). Impact of lipoprotein (a) on long-term outcomes in patients with acute myocardial infarction. Circ. J..

[40] O'Donoghue M.L., Fazio S., Giugliano R.P., Stroes E.S.G., Kanevsky E., Gouni-Berthold I., Im K., Lira Pineda A., Wasserman S.M., Ceska R. (2019). Lipoprotein(a), PCSK9 inhibition, and cardiovascular risk. Circulation.

[41] Dordonne S., Mergeayfabre M., Hafsi N., Ntoutoum A., Salazar-Cardozo C., Casse O., Hounnou M., Adenis A., Aurelus J.M., Misslin-Tristch C. (2023). Impact of lipoprotein(a) on macrovascular complications of diabetes in a multiethnic population in the French amazon. J. Diabetes Res..

