# Supplementary material for: Correlation between circulating lipoprotein(a) levels and cardiovascular events risk in patients with type 2 diabetes
Source: Heliyon. 2024 Sep 4;10(17):e37415. doi: 10.1016/j.heliyon.2024.e37415 (PMC11408807; doi:10.1016/j.heliyon.2024.e37415)
Supplement: Multimedia component 1 [file mmc1.docx]

Supplementary table 1 The definition of MI, stroke, heart failure, unstable angina, three-vessel disease and Gensini score.

| **Variables** | **Definition** | | | |
| --- | --- | --- | --- | --- |
| **MI** | • Acute myocardial infarction  • Myocardial infarction  • Myocardial infarction (SMQ, broad and narrow) | | | |
| **Stroke** | • Cerebral infarction  • Haemorrhagic stroke  • Ischaemic stroke  • Lacunar stroke  • Cerebrovascular accident | | | |
| **Heart failure** | • Heart failure with reduced ejection fraction   - Heart failure with preserved ejection fraction | | | |
| **Unstable angina** | • Rest angina  • New-onset angina  • Accelerating angina | | | |
| **three-vessel disease** | • Right coronary artery  • Left anterior descending coronary artery  • Left coronary artery circumflex branch | | | |
| **Gensini score** | **Obstruction degree** | **score** | **Lesion site** | **score** |
|  | 1 %～25 % | 1 | Left main trunk | ×5 |
|  | 26 %～50 % | 2 | Left anterior descending branch or proximal segment of circumflex branch | ×2.5 |
|  | 51 %～75 % | 4 | Middle section of left anterior descending branch | ×1.5 |
|  | 76 %～90 % | 8 | Left anterior descending branch distal segment | ×1 |
|  | 91 %～99 % | 16 | Middle and distal segments of the left circumflex branch | ×1 |
|  | 100% | 32 | Right coronary artery | ×1 |
|  |  |  | Small branch | ×0.5 |
| The score for each lesion is calculated by multiplying the stenosis score by the lesion score, and the score for each patient is the sum of all lesion scores. | | | | |
