# Supplementary material for: Correlation between circulating lipoprotein(a) levels and cardiovascular events risk in patients with type 2 diabetes
Source: Heliyon. 2024 Sep 4;10(17):e37415. doi: 10.1016/j.heliyon.2024.e37415 (PMC11408807; doi:10.1016/j.heliyon.2024.e37415)
Supplement: Multimedia component 2 [file mmc2.docx]

Supplementary Table 2. Baseline characteristics of type 2 diabetic patients with coronary heart disease at different Lp(a) levels

| **Variables** | **Lp(a) concentration (nmol/L)** | | | ***p* value** |
| --- | --- | --- | --- | --- |
|  | Low Lp(a) | Mid Lp(a) | High Lp(a) |  |
|  | < 32.37 | 32.37 – 57.58 | > 57.58 |  |
| Clinical characteristics |  |  |  |  |
| N (%) | 831 (33.31 %) | 834 (33.40%) | 832 (33.29 %) | – |
| Age (years) | 54.19 ± 10.91 | 55.91 ± 10.11 | 54.78 ± 11.16 | 0.295 |
| Male (%) | 335 (40.31 %) | 355 (42.57%) | 343 (41.23 %) | 0.643 |
| BMI (kg/m^2^) | 24.65 ± 3.08 | 24.28 ± 2.84 | 24.75 ± 2.77 | 0.471 |
| Hypertension (%) | 588 (70.76%) | 598 (71.70%) | 624 (75.00%) | 0.126 |
| Smoking (%) | 245 (29.48%) | 226 (27.10%) | 225 (27.04%) | 0.448 |
| Consumers of alcohol (%) | 270 (32.49%) | 259 (31.06%) | 275 (33.05%) | 0.667 |
| Family history of CHD (%) | 251 (30.20%) | 245 (29.38%) | 276 (33.17%) | 0.212 |
| Family history of MD (%) | 365 (43.92%) | 350 (41.97%) | 348 (41.83%) | 0.627 |
| Blood sugar is well controlled (%) | 570 (68.59%) | 546 (65.47%) | 581 (69.83%) | 0.144 |
| Duration of diabetes > 5 years (%) | 201 (24.19%) | 179 (21.46%) | 171 (20.55%) | 0.177 |
| Diabetic eye disease (%) | 154 (18.53%) | 160 (19.18%) | 184 (22.12%) | 0.150 |
| Diabetic neuropathy (%) | 234 (28.16%) | 238 (28.54%) | 251 (30.17%) | 0.631 |
| Statin drug therapy (%) | 758 (91.22%) | 779 (93.41%) | 761 (91.47%) | 0.196 |
| Laboratory variables |  |  |  |  |
| FPG (mmol/L) | 7.48 ± 1.09 | 7.53 ± 1.26 | 7.58 ± 1.23 | 0.716 |
| HbA_1c_ (%) | 7.3 ± 0.9 | 7.2 ± 1.1 | 7.2 ± 1.1 | 0.753 |
| ApoB (mg/dL) | 75.20 (31.74) | 90.44 (35.70)^a^ | 100.64 (38.74)^ab^ | <0.001 |
| ApoA1 (mg/dL) | 140.5 (46.10) | 139.80 (42.35) | 135.60 (39.70) | 0.197 |
| Total cholesterol (mmol/L) | 3.99 (1.48) | 4.51 (1.55)^a^ | 4.87 (1.77)^ab^ | <0.001 |
| Triglycerides (mmol/L) | 1.25 (0.80) | 1.33 (0.87) | 1.35 (0.77) | 0.636 |
| HDL-C (mmol/L) | 1.08 (0.37) | 1.07 (0.29) | 1.02 (0.26) | 0.053 |
| LDL-C (mmol/L) | 2.46 (1.17) | 2.77 (1.27)^a^ | 3.10 (1.34)^ab^ | <0.001 |
| hs-CRP (mg/L) | 1.42 (1.68) | 1.50 (1.80) | 1.76 (1.69) | 0.355 |
| HCY (umol/L) | 9.96 (6.35) | 10.74 (7.13) | 13.90 (10.18)^ab^ | <0.001 |
| sdLDL-C (mmol/L) | 0.67 (0.43) | 0.78 (0.44)^a^ | 0.86 ( 0.50)^a^ | <0.001 |
| Lp(a) (nmol/L) | 23.39 (8.95) | 41.71 (11.80) | 97.65 (65.54) | – |
| Diseased vessels in CHD patients |  |  |  |  |
| Single-vessel (%) | 396 (47.65%) | 181 (21.70%)^a^ | 166 (19.95%)^a^ | <0.001 |
| Double-vessels (%) | 247 (29.72%) | 286 (34.29%)^a^ | 243 (29.21%)^b^ | 0.041 |
| Triple-vessels (%) | 188 (22.62%) | 367 (44.00%)^a^ | 423 (50.84%)^ab^ | <0.001 |
| Gensini score | 26 (22) | 33 (23)^a^ | 39 (23)^ab^ | <0.001 |

Data are reported as means ± SD or n(%), median (interquartile ranges). SD: Standard deviation

BMI: body mass index; CHD: coronary heart disease; DM: diabetes mellitus; FPG: fasting plasma glucose; HbA1c: Hemoglobin A1c; apoB: apolipoprotein B; apoA1: apolipoprotein A1; HDL-C: high density lipoprotein cholesterol; LDL-C: low density lipoprotein cholesterol; Hs-CRP: hypersensitive C-reactive protein; HCY: homocysteine; sdLDL-C: small dense low-density lipoprotein cholesterol; Lp(a): lipoprotein (a).

Statistical analysis was performed with the ANOVA or Kruskal – Wall test and with Chi-square test for categorical variables.

a: Shows that the p < 0.05 compared with the Low Lp(a) group.

b: Shows that the p < 0.05 compared with the Mid Lp(a) group.
