# Supplementary material for: Correlation between circulating lipoprotein(a) levels and cardiovascular events risk in patients with type 2 diabetes
Source: Heliyon. 2024 Sep 4;10(17):e37415. doi: 10.1016/j.heliyon.2024.e37415 (PMC11408807; doi:10.1016/j.heliyon.2024.e37415)
Supplement: Multimedia component 3 [file mmc3.docx]

Supplementary table 3. The relationship between Lp(a) level and the MACEs outcomes in Non-CHD group

| **MACEs** | **Lp(a) concentration (nmol/L)** | | | ***p*** | |
| --- | --- | --- | --- | --- | --- |
|  | Low Lp(a) | Mid Lp(a) | High Lp(a) | |  |
|  | < 31.51 | 31.51 – 53.58 | > 53.58 | |  |
|  | n = 715 | n = 611 | n = 509 | |  |
| Cardiovascular deaths | 1 (0.14%) | 3 (0.49%) | 3 (0.60%) | | 0.329 |
| Non-fatal MI | 16 (2.24%) | 19 (3.11%) | 22 (4.32%) | | 0.058 |
| Non-fatal strokes | 10 (1.40%) | 12 (1.96%) | 14 (2.75%) | | 0.149 |
| Heart failure | 10 (1.40%) | 11 (1.80%) | 13 (2.55%) | | 0.217 |
| Hospitalization for unstable angina | 16 (2.24%) | 15 (2.45%) | 18 (3.54%) | | 0.222 |
| Total | 53 (7.41%) | 60 (9.82%) | 70 (13.75%)^ab^ | | < 0.001 |

Lp(a): lipoprotein (a); CHD: Coronary heart disease; MACEs: major adverse cardiovascular events; MI: myocardial infarction.

Statistical analysis was performed with Chi-square test for categorical variables.

a: Shows that the *p* < 0.05 compared with the Low Lp(a) group.

b: Shows that the *p* < 0.05 compared with the Mid Lp(a) group.
