# Supplementary material for: Correlation between circulating lipoprotein(a) levels and cardiovascular events risk in patients with type 2 diabetes
Source: Heliyon. 2024 Sep 4;10(17):e37415. doi: 10.1016/j.heliyon.2024.e37415 (PMC11408807; doi:10.1016/j.heliyon.2024.e37415)
Supplement: Multimedia component 4 [file mmc4.docx]

Supplementary table 4. The relationship between Lp(a) level and the MACEs outcomes in CHD group

| **MACEs** | **Lp(a) concentration (nmol/L)** | | | ***p*** |
| --- | --- | --- | --- | --- |
|  | Low Lp(a) | Mid Lp(a) | High Lp(a) |  |
|  | < 31.51 | 31.51 – 53.58 | > 53.58 |  |
|  | n = 728 | n = 836 | n = 933 |  |
| Cardiovascular deaths | 4 (0.55%) | 6 (0.72%) | 10 (1.07%) | 0.347 |
| Non-fatal MI | 23 (3.16%) | 36 (4.31%) | 60 (6.43%)^ab^ | 0.002 |
| Non-fatal strokes | 17 (2.34%) | 28 (3.35%) | 38 (4.07%) | 0.064 |
| Heart failure | 15 (2.06%) | 26 (3.11%) | 34 (3.64%) | 0.080 |
| Hospitalization for unstable angina | 21 (2.88%) | 34 (4.07%) | 59 (6.32%)^ab^ | 0.001 |
| Total | 80 (10.99%) | 130 (15.55%)^a^ | 201 (21.54%)^ab^ | < 0.001 |

Lp(a): lipoprotein (a); CHD: Coronary heart disease; MACEs: major adverse cardiovascular events; MI: myocardial infarction.

Statistical analysis was performed with Chi-square test for categorical variables.

a: Shows that the *p* < 0.05 compared with the Low Lp(a) group.

b: Shows that the *p* < 0.05 compared with the Mid Lp(a) group.
