# Supplementary material for: Correlation between circulating lipoprotein(a) levels and cardiovascular events risk in patients with type 2 diabetes
Source: Heliyon. 2024 Sep 4;10(17):e37415. doi: 10.1016/j.heliyon.2024.e37415 (PMC11408807; doi:10.1016/j.heliyon.2024.e37415)
Supplement: Multimedia component 5 [file mmc5.docx]

Supplementary table 5. The relationship between Lp(a) level and the MACEs outcomes in all patients

| **MACEs** | **Lp(a) concentration (nmol/L)** | | | ***p*** | |
| --- | --- | --- | --- | --- | --- |
|  | Low Lp(a) | Mid Lp(a) | High Lp(a) | |  |
|  | < 31.51 | 31.51 – 53.58 | > 53.58 | |  |
|  | n = 1443 | n = 1447 | n = 1442 | |  |
| Cardiovascular deaths | 5 (0.35%) | 9 (0.62%) | 13 (0.90%) | | 0.105 |
| Non-fatal MI | 39 (2.70%) | 55 (3.80%) | 82 (5.69%)^ab^ | | < 0.001 |
| Non-fatal strokes | 27 (1.87%) | 40 (2.76%) | 52 (3.61%)^a^ | | 0.005 |
| Heart failure | 25 (1.73%) | 37 (2.56%) | 47 (3.26%)^a^ | | 0.010 |
| Hospitalization for unstable angina | 37 (2.56%) | 49 (3.39%) | 77 (5.34%)^ab^ | | < 0.001 |
| Total | 133 (9.22%) | 190 (13.13%)^a^ | 271 (18.79%)^ab^ | | < 0.001 |

Lp(a): lipoprotein (a); CHD: Coronary heart disease; MACEs: major adverse cardiovascular events; MI: myocardial infarction.

Statistical analysis was performed with Chi-square test for categorical variables.

a: Shows that the *p* < 0.05 compared with the Low Lp(a) group.

b: Shows that the *p* < 0.05 compared with the Mid Lp(a) group.
